# Unintended Pregnancy among Pregnant Women in Ghana: Prevalence and Predictors

**DOI:** 10.1155/2019/2920491

**Published:** 2019-01-30

**Authors:** Samuel H. Nyarko

**Affiliations:** Department of Demography, College of Public Policy, The University of Texas at San Antonio, San Antonio, TX, USA

## Abstract

**Background:**

Unintended pregnancy is seen as the key concept for better understanding the fertility and the unmet need for family planning of populations. It is seen as a major challenge among women in many developing countries including Ghana. However, there is scarcely nationally representative information on its prevalence and predictors in Ghana.

**Methods:**

In a cross-sectional study design, data for this study were extracted from the 2014 Ghana Demographic and Health Survey. The prevalence of unintended pregnancies was computed, and logit regression models were fitted to predict the factors influencing unintended pregnancies in the country.

**Results:**

The total prevalence of unintended pregnancies among pregnant women in Ghana was found to be 40%. Background characteristics such as age (OR=4.85, CI=1.48-15.84), level of education (OR=0.50, CI=0.26-1.01), marital status (OR=3.83, CI=1.67-8.75), parity (OR=0.13, CI=0.05-0.32), and region of residence (OR=0.11, CI=0.03-0.31) were the significant predictors of unintended pregnancy, net of unmet need for contraception. However, unmet need for contraception (OR=7.13, CI=1.57-8.91) serves as an independently significant predictor of unintended pregnancy regardless of the background characteristics of respondents.

**Conclusions:**

The study findings strongly underscore the need for significant improvement in the access to contraception methods and family planning information in the quest to considerably reduce unintended pregnancies in the entire country.

## 1. Background

An unintended pregnancy has been defined as the kind of pregnancy that is reported to be either unwanted or mistimed [1]. Unintended pregnancy is regarded as the principal concept for understanding the fertility and the unmet need for contraception and family planning of populations. It usually occurs as a result of nonuse and inconsistent or incorrect use of effective contraceptive methods [1]. Globally, it is estimated that about 85 million pregnancies, representing 40 percent of all pregnancies, were unintended in 2012, of which 50 percent ended in abortion, 13 percent ended in miscarriage, and 38 percent resulted in an unplanned birth [2]. Even though the incidence of unintended pregnancy has declined globally in the past decade, the rate of unintended pregnancy remains high, particularly in developing regions such as sub-Saharan Africa [3].

In Ghana, it has been identified that about 37 percent of all pregnancies are unintended, comprising 23 percent mistimed and 14 percent unwanted pregnancies [4]. Consequently, thousands of pregnancies are aborted while more than 300,000 infants are born as a result of unintended pregnancies in Ghana [5]. These levels are believed to be still high albeit the trends have been waning for the past decades. It is believed that poverty, stigmatisation of unmarried mothers, a cultural preference for sons, competing demands on women's time, and poor access to family planning services among many others are the underlining causes of unintended pregnancy [3].

Unintended pregnancy has been found to have severe implications for both the child and the mother. For instance, unintended pregnancy is found to have negative effects on prenatal visits and care, physical health status, labour experience, pain during labour, and psychological status in the early postpartum period [6]. It is also found to have negative effects on various aspects of maternal and child health and behaviour [7], as well as increased risks of maternal depression and parenting stress. Unintended pregnancies are also known to impose astronomical direct and indirect financial costs on individuals and couples as well as governments [8–10].

Even though myriads of extant studies have examined the levels and determinants of unintended pregnancy globally in the past few decades, information on this phenomenon is quite limited in Ghana. To the best of my knowledge, the few extant studies are clinic-based and focused on only the rural population [11, 12]; hence, their findings may not represent the entire country. However, this study provides findings that are nationally representative. It, therefore, becomes important to ask the following: what is the level of unintended pregnancy prevalence among pregnant women in Ghana; what are the factors that predict unintended pregnancy among pregnant women in Ghana? These are the pertinent research questions that this study sought to answer.

## 2. Materials and Methods

### 2.1. Study Design and Data Source

This is a cross-sectional study that used data from the 2014 Ghana Demographic and Health Survey (GDHS). The 2014 GDHS is the sixth round of the global DHS program series, which was conducted by the Ghana Statistical Service (GSS) and the Ghana Health Service (GHS) and was financially supported by the United States Agency for International Development (USAID) among other organisations. It is a nationally representative survey and it comprised 9,396 women aged 15-49 who had been selected from 11,835 households using a two-stage sampling design [13]. The survey provides a complete birth history of women; as a result, it serves as the best source of data for this study. The unit of analysis is individual pregnant women at the time of the survey (679); as a result, only the data concerning them were extracted for this study. Ethical approval and consent to participate were provided by the Ghana Health Service; consequently, this is not applicable to this study.

### 2.2. Study Variables

The outcome variable for this study is named “current pregnancy wanted” in the dataset. In this regard, women who participated in the survey were asked whether they were currently pregnant and those who responded in the affirmative were further asked whether they wanted this pregnancy. This generated three responses such as “wanted then,” “wanted later,” and “not at all.” To generate the outcome variable for unintended pregnancy, a dichotomous outcome was created by recoding the original variable “wanted then” as “intended(0)” and “wanted later” and “not at all” as “unintended(1)”. Twelve sociodemographic predictor variables were used in the analysis, which comprised the age of respondent, education level, religion, ethnicity, wealth status, marital status, parity, work status, and knowledge of ovulatory cycle, including control variables such as the type and region of residence and unmet need for contraception. These predictor variables have been included in this study primarily based on their effect in the extant literature concerning the subject.

### 2.3. Analytical Strategy

The R programming language (version 3.4.3) was used to process the data. The data were imported from the GDHS Stata file into R. Descriptive and bivariate analyses were performed to ascertain the level of unintended pregnancy prevalence among the predictor variables. Furthermore, logit regression models were fitted to predict the determinants of unintended pregnancy among Ghanaian women. A logit regression model was chosen because it is suitable for binary outcomes and provides the opportunity to estimate the risk factors of a phenomenon while also providing the opportunity to generate odds ratios for them. Three logit models were fitted where model 1 examines the sociodemographic predictors of unintended pregnancy whereas model 2 examines whether the sociodemographic predictors may complement spatial factors such as type and region of residence to predict unintended pregnancy by nesting model 1 within model 2. Model 3 was used to control for exposure to the need for contraception. A complex survey design was applied to the results to produce nationally representative findings. In doing this, a survey design was generated where the primary sampling units, the strata, and the weight were nested in the final dataset used for the analysis.

## 3. Results

### 3.1. Background Characteristics of Respondents

This study comprised pregnant women at the time of the survey and a summary of their background characteristics is presented in Table 1. As indicated in the table, 44.3% were aged 20-29, and about 41% were aged 30-39 while only 5.4% were aged 40 to 49. About 60% had secondary school or higher education while only 16.4% had primary school education. The majority were Christians (79.8%) while the least comprised women from other miscellaneous affiliations (2.3%). For ethnicity, more than half of the respondents were Akans (51.0%) while just a few were Ga-Dangmes (4.4%). Also, 42.8% were from rich households while more than one-third were from poor households (36.6%).

The majority of the respondents were married or living together (82.9%) whereas the least were widowed, separated, or divorced (1.8%). More than half had 1-3 children (55.9%) while more than one-fifth had no children (21.2%). The majority were working (74.6%) while the minority were not working (25.4%). Also, more than half did not know their ovulatory cycle (57.8%) while 42.2% knew their ovulatory cycle. Furthermore, 60.0% of the respondents had no unmet need for contraception while 40.0% had unmet need for contraception. A little more than half of the respondents were from rural settings (50.3%) while the remaining women were from urban settings (49.7%). Lastly, about one-fifth of the respondents were from the Greater Accra (19.7%), 15.7% were from the Ashanti region, and 11.1% were from the Central region while only 2.2% were from the Upper East region.

### 3.2. Prevalence of Unintended Pregnancy among Respondents

Table 1 shows the results of the prevalence of unintended pregnancy across the background characteristics of the respondents. The total prevalence of unintended pregnancy among the respondents was 40%. The prevalence of unintended pregnancy was highest among respondents aged 15-19 (71.7%) and 40-49 (50.2%) than their counterparts who were in their thirties. The prevalence was also higher among respondents who had primary school education (53.9%) and secondary school or higher education (41.6%) than those who had no formal education. Also, unintended pregnancy was more prevalent among traditionalists (44.1%) and Christians (43.3%) and other women (42.7%) than Muslim women. Furthermore, the prevalence was highest among Ewe women (60.0%) and Akan women (43.1%) but was lowest among Other women (23.4%). Middle class (48.5%) and poor women (44.0%) had higher prevalence of unintended pregnancy than women from rich households.

Women who were never married (68.9%) also had the highest prevalence of unintended pregnancy while close to half of widowed, separated or divorced women (48.2%) had an unintended pregnancy, with married or women in union (34.4%) having the lowest. Additionally, women who had 4 or more (53.3%) children had the highest prevalence with more than half of them having unintended pregnancy while women having 1-3 children (31.9%) had the lowest. Nonworking women (47.7%) also had a higher prevalence of unintended pregnancy than working women (37.4%) whereas women who knew their ovulatory cycle (39.2%) and women who did not know (40.0%) almost had the same prevalence of unintended pregnancy. Quite expectedly, all the women who had unmet need for contraception (100.0%) had unintended pregnancy whereas none of the women who had no unmet need for contraception had any unintended pregnancy.

In addition, unintended pregnancies were more prevalent among rural residents (46.7%) than urban residents (33.1%). With regard to variations in regional prevalence, unintended pregnancies were more prevalent in the Volta region (76.2%), Eastern region (56.6%), and the Central region (51.5%) compared to the other regions in the country, but lowest in the Northern region (12.3%).

### 3.3. Predictors of Unintended Pregnancy among Respondents

In this section, I assess the predictors of unintended pregnancy among women using three nested logit models, and the results are presented in Table 2. The results show that there is a significant relationship between unintended pregnancy and age of respondent for model 1 and model 2 after controlling for spatial factors. However, this effect has disappeared once the unmet need for contraception was controlled in model 3. From model 2, it can be observed that the odds of unintended pregnancy were higher for respondents aged 20-29 (3.62 times, CI=1.56-8.34), 30-39(6.59 times, CI=2.56-16.73), and 40-49 (4.85 times, CI=1.48-15.84) compared to respondents aged 15-19. In both models 1 and 2, the results show a positive but curvilinear relationship with age where the likelihood of unintended pregnancy reached its peak at ages 30-39 and started to wane from the age of 40.

Also, respondent's education level had a significant and negative relationship with unintended pregnancy in models 1 and 2 when spatial factors were controlled, but these disappeared when the unmet need for contraception was controlled. The odds of unintended pregnancy were 49% lower for respondents with primary school education (CI=0.25-0.98) and 50% lower for respondents with secondary school education or higher (CI=0.26-1.01) compared to respondents without any formal education. However, religious affiliation had no significant relationship with unintended pregnancy in all the models, though Muslim women and Others had higher odds of unintended pregnancy compared to the Christians. Similarly, wealth status had no significant effect on unintended pregnancy in all the models. The ethnic background of the respondent was found significant in only model 1, with Ewe respondents having 58% lower odds whereas Other ethnic minorities had 129% higher odds compared to Akan respondents.

Additionally, there was a significant relationship between marital status and unintended pregnancy for the first two models, but the significance disappeared in the third model. The odds of unintended pregnancy were higher for respondents who were married or cohabiting (3.83 times, CI=1.67-8.75) compared to respondents who had never been married or in a union. The parity of the respondents also shows a significant relationship with unintended pregnancy, with women who had 4 or more children significantly having 87% lower odds compared to women who had no children. However, work status, knowledge of ovulatory cycle, and type of residence show no significant effect on the risk of unintended pregnancy across all the models.

Furthermore, the results show that the region of residence had a significant effect on unintended pregnancy in model 2 but not in model 3, after controlling for unmet need for contraception. Respondents from regions such as Central (OR=0.33, CI=0.10-0.90), Greater Accra (OR=0.33, CI=0.08-0.61), Volta (OR=0.11, CI=0.03-0.31), Eastern (OR=0.22, CI=0.10-0.50), and Ashanti region (OR=0.21, CI=0.09-0.52) all had significantly lower odds of having unintended pregnancy compared to their counterparts from the Western region. Ultimately, in model 3, only unmet need for contraception had a significant effect on unintended pregnancy with those having unmet need having 7.13 odds of unintended pregnancy (CI=1.57-8.91) compared to those without an unmet need for contraception.

## 4. Discussion

One of the objectives of this study was to determine the prevalence of unintended pregnancy among women in Ghana. This study also sought to establish the factors that predict unintended pregnancies among women in Ghana. The results show that the prevalence of unintended pregnancy among women in Ghana is excessively high. About 40% of pregnant women reported that they either wanted the pregnancy later or did not want it at all. This is just in the range of the prevalence found by other studies [14]. Also, there were substantial disparities in the prevalence of unintended pregnancy across the various categories of the background characteristics. For instance, the prevalence of unintended pregnancy was considerably higher for women aged 15-19 (71.7%) compared to that of women aged 30-39 (34.7%). In a systematic review of various studies, it was established that the prevalence of unintended pregnancy ranged from 13% to 82% with an average of 35% [6, 9, 10, 15].

In examining the factors that influence the high level of unintended pregnancy among pregnant women in Ghana, the study found some sociodemographic characteristics to be significantly associated with unintended pregnancy, net of unmet need for contraception. The age of the woman is found to have predicted the risk of unintended pregnancy among pregnant women in Ghana, net of unmet need for contraception. The relationship between age and unintended pregnancy is curvilinear where the risk of unintended pregnancy increases steadily with age until it reaches its peak among women aged 30-39, before waning among women in their 40s. Thus, even though the prevalence of unintended pregnancy was highest among women aged 15-19, the risk of unintended pregnancy is actually higher among older women in Ghana. This is consistent with the findings of extant studies which also found age to be a significant determinant of unintended pregnancy [14, 16, 17]. Older Ghanaian women may be at higher risk of unintended pregnancy because they are most likely to have attained their desired number of children and, therefore, may not want any more children.

The results further show that unintended pregnancy is significantly determined by the education level of the Ghanaian woman. The effect of educational attainment on unintended pregnancy is quite evident in the extant literature [18–20]. This finding shows a negative relationship between the risk of unintended pregnancy and education level among Ghanaian women. Consequently, highly educated women have a considerably lower risk of unintended pregnancy. This places Ghanaian women without formal education at an extreme disadvantage with regard to the risk of unintended pregnancy. The likely reason may be that Ghanaian women with some formal education or who are highly educated are more empowered to take control of their sexual and reproductive health matters than their counterparts who have no formal education.

According to the findings of this study, marital status also plays a significant role in the risk of unintended pregnancy among Ghanaian women independent of the unmet need for contraception. It is evident that married Ghanaian or cohabiting women are substantially more likely to be at risk of unintended pregnancy than those who never married and those who are previously married. In this context, the higher risk of unintended pregnancy among Ghanaian married women may be due to nonuse of contraceptives or contraceptive failure as many Ghanaian women may believe that contraceptive use has serious side effects while some may also believe that it is a sin. The significance of the marital status and unintended pregnancy nexus is also established by Ikamari et al. [16], Hall et al. [18], and Exavery et al. [21], among others. However, unlike this study, some of these studies instead found a higher risk of unintended pregnancy among unmarried women.

Additionally, the risk of unintended pregnancy is found to be predicted by the parity of the Ghanaian woman, net of unmet need for contraception. In this study, higher parity women unexpectedly have a significantly lower risk of unintended pregnancy compared to those with lower parity. In context, this may be a consequence of uptake of family planning programmes among this category of Ghanaian women who might have decided to stop childbirth. On the contrary, even though many studies have documented the effect of parity on the risk of unintended pregnancy [12, 20, 22], virtually all these studies show that the higher risk of unintended pregnancy is instead among higher parity women.

Moreover, the region of residence is significantly associated with unintended pregnancy when the unmet need for contraception is not controlled. In effect, women in the Central, Greater Accra, Volta, Eastern, and the Ashanti region have a significantly lower risk of unintended pregnancy than their counterparts from the other regions. These are virtually southern sector regions of Ghana where much of the socioeconomic development is found; therefore, women in these regions are most likely exposed to family planning facilities and services compared to their counterparts from the northern sector. Women from the Northern region and the Upper East region are at higher risk of unintended pregnancy in the country. It is noteworthy that the role of the region of residence in the risk of unintended pregnancy is not well-documented in the literature. However, a few studies have observed the significance of region of residence as a determinant of unintended pregnancy among women [17].

Ultimately, this study shows that net of background characteristics such as age, education, and marital status, among others, unmet need for contraception independently has a significant effect on the risk of unintended pregnancy among Ghanaian women. This is evident in model 3 where the significance of all significant factors disappeared after controlling for unmet need for contraception. The implication is that, irrespective of the background characteristics, unmet need for contraception is a principal predictor of unintended pregnancy among Ghanaian women. In this regard, women who have an unmet need for contraception in Ghana have a significantly higher risk of unintended pregnancy than those without an unmet need for contraception. This significant positive relationship between unmet need for contraception and unintended pregnancy is consistent with extant literature [23, 24]. Finally, one potential caution about this study is that it is based on a single wave of a cross-sectional survey; therefore, the findings reflect the situation of a point in time and may change over time. However, these findings make an indispensable contribution to knowledge on the risk of unintended pregnancy in Ghana.

## 5. Conclusions

This study provides evidence that the prevalence of unintended pregnancy among pregnant women in Ghana is excessively high. This is significantly predicted by a number of background characteristics such as the age of the woman, educational attainment, marital status, parity and region of residence, independent of unmet need for contraception. However, unmet need for contraception shows a significant independent effect on the risk of unintended pregnancy among Ghanaian women, irrespective of their background characteristics. The older, the uneducated, the married or cohabiting, and the zero parity women, as well as women in the Western and some Northern regions, all have a higher risk of unintended pregnancy in the country. Public policy decisions must be focused on substantially reducing unmet need for contraception among Ghanaian women. Using the background risk factors as a basis, unmet need for contraception can be tackled by greatly improving access to all forms of contraception methods and family planning services among both Ghanaian women and men. Also, policy options should be considered to tackle and reduce any attitudinal resistance to effective contraceptive use among Ghanaian women. These would help to improve effective contraceptive use and prevent many unwanted pregnancies among Ghanaian women. Unlike this study, future research can focus on including both individual and aggregate level factors that have an effect on the risk of unintended pregnancy in Ghana including the contextual factors.

## Figures and Tables

**Table 1:** Sociodemographic characteristics and prevalence of unintended pregnancy.

Variables	Number of women	Percent	Unintended pregnancy
Age			(%)
15-19	64	9.4	71.7
20-29	301	44.3	36.9
30-39	278	40.9	34.7
40-49	36	5.4	50.2
Education level			
No education	161	23.8	26.1
Primary education	113	16.6	53.9
Secondary/higher	405	59.6	41.6
Religious affiliation			
Christian	542	79.8	43.3
Muslim	102	15.1	21.3
Traditionalist	19	2.8	44.1
Others	16	2.3	42.7
Ethnicity			
Akan	346	51.0	43.1
Ga-Dangme	30	4.4	35.7
Ewe	109	16.1	60.0
Mole-Dagbani	99	14.6	24.0
Others	95	13.9	23.4
Wealth status			
Poor	248	36.6	44.0
Middle	140	20.6	48.5
Rich	291	42.8	32.4
Marital status			
Never married	104	15.3	68.9
Married/living together	563	82.9	34.4
Widowed/divorced/separated	12	1.8	48.2
Parity			
0	144	21.2	46.8
1-3	380	55.9	31.9
4+	155	22.9	53.3
Work status			
Working	507	74.6	37.4
Not working	172	25.4	47.5
Ovulatory cycle knowledge			
Yes	287	42.2	39.8
No	392	57.8	40.0
Unmet need for contraception			
No unmet need	408	60.0	000.0
Unmet need	271	40.0	100.0
Type of residence			
Urban	337	49.7	33.1
Rural	342	50.3	46.7
Region			
Western	73	10.7	27.6
Central	75	11.1	51.5
Greater Accra	134	19.7	36.9
Volta	45	6.6	76.2
Eastern	71	10.4	56.6
Ashanti	106	15.7	46.6
Brong Ahafo	60	8.8	36.9
Northern	71	10.5	12.3
Upper East	29	4.3	16.9
Upper West	15	2.2	23.6
Total prevalence			40.0

**Table 2:** Logit regression analysis of unintended pregnancy among pregnant women.

**Variables**	**Model 1**	**Model 2**	**Model 3**
Age of woman	**OR [CI]**	**OR [CI]**	**OR [CI]**
15-19 (Ref)	1		
20-29	3.42[1.27, 9.22]*∗∗*	3.62[1.56, 8.34]*∗∗∗*	1.02[0.68, 1.51 ]
30-39	5.81[2.04,16.55]*∗∗∗*	6.59 [2.59, 16.73]*∗∗∗*	1.05[0.67, 1.63]
40-49	4.58[1.21, 15.96]*∗∗*	4.85[1.48, 15.84]*∗∗∗*	1.04[0.59, 1.81]
Level of education			
No education (Ref)	1		
Primary	0.44[0.20, 0.94]*∗∗*	0.51[0.25, 0.98]*∗*	0.95[0.73, 1.35]
Secondary/higher	0.50[0.25, 0.99]*∗∗*	0.50[0.26, 1.01]*∗*	0.76[0.73, 1.29]
Religious affiliation			
Christianity (Ref)	1		
Islam	1.47[0.72, 2.97]	1.46[0.74, 2.89]	0.96[0.69, 1.35]
Traditional/	0.52[0.14, 1.91]	0.33[0.06, 1.67]	1.06[0.61, 1.82]
Spiritual			
Other	1.16[0.28, 4.77]	1.14[0.30, 4.31]	0.97[0.52, 1.78]
Ethnicity			
Akan (Ref)	1		
Ga/Dangme	1.07[0.41, 2.77]	1.44[0.49, 4.18]	1.04[0.60, 1.79]
Ewe	0.42[0.25, 0.73]*∗∗∗*	0.68[0.32, 1.47]	0.94[0.64, 1.36]
Mole-Dagbani	1.64[0.80, 3.36]	0.77[0.33, 1.79]	0.98[0.67, 1.45]
Other	2.29[1.50, 7.24]*∗∗*	1.11[0.53, 2.31]	0.98[0.68, 1.40]
Wealth status			
Poor (Ref)	1	1	
Middle	0.88[0.52, 1.51]	0.86[0.48, 1.55]	1.04[0.78, 1.32]
Rich	1.65[0.95, 2.84]	1.65[0.81, 3.37]	0.96[0.70, 1.33 ]
Marital status			
Never married (Ref)	1		
Married/Cohabiting	3.30[1.50, 7.24]*∗∗∗*	3.83[1.67, 8.75]*∗∗∗*	1.01[0.71, 1.43]
Widowed/divorced/Separated	2.18[0.50, 9.58]	1.69[0.33, 8.62]	0.98[0.46, 2.06]
Parity			
0 (Ref)	1		
1-3	0.64[0.31, 1.32]	0.56[0.26, 1.18]	0.98[0.74, 1.30]
4+	0.15[0.06, 0.35]*∗∗∗*	0.13[0.05, 0.32]*∗∗∗*	0.95[0.64, 1.39]
Work status			
Working (Ref)	1		
Not working	0.81[0.49, 1.34]	0.75[0.44, 1.28]	1.01[0.81, 1.25]
Ovulatory cycle knowledge			
Yes	1		
No	1.03[0.66, 1.59]	1.02[0.64, 1.61]	1.01[0.83, 1.22]
Type of residence			
Urban (Ref)		1	
Rural		0.63[0.35, 1.12]	0.97[0.75, 1.24]
Region of residence			
Western		1	
Central		0.30[0.10, 0.90]*∗∗*	0.95[0.60, 1.50]
Greater Accra		0.22[0.08, 0.61]*∗∗∗*	1.09[0.74, 1.61]
Volta		0.11[0.03, 0.31]*∗∗∗*	1.03[0.63, 1.69]
Eastern		0.22[0.10, 0.50]*∗∗∗*	0.97[0.68, 1.38]
Ashanti		0.21[0.09, 0.52]*∗∗∗*	0.99[0.7, 1.37]
Brong Ahafo		0.46[0.17, 1.19]	0.98[0.68, 1.42]
Northern		2.12[0.75, 5.98]	0.89[0.58, 1.37]
Upper East		1.63[0.45, 5.79]	0.84[0.47, 1.49]
Upper West		0.83[0.24, 2.81]	0.82[0.53, 1.26]
Unmet need for contraception			
No unmet need (Ref)			1
Unmet need			7.13[1.57, 8.91]*∗∗∗*

Log Likelihood	-395.411	-367.130	-311.281
AIC	832.822	796.259	764. 463

Note: OR=odds ratios; CI= confidence intervals; Ref= reference category. *∗*p≤0.05; *∗∗*p≤0.01; *∗∗∗*p≤0.001.

## Data Availability

The dataset used to support the findings of this study is available at the DHS Program data repository.
